# Supplementary material for: Extent of Mucosal Inflammation in Ulcerative Colitis Influences the Clinical Remission Induced by Vedolizumab
Source: J Clin Med. 2020 Feb 1;9(2):385. doi: 10.3390/jcm9020385 (PMC7073927; doi:10.3390/jcm9020385)
Supplement: Supplementary file 1 [file jcm-09-00385-s001.pdf]

Table S1. Distribution of the clinical activity of disease at baseline in Crohn's disease patients with ileal disease (L1), colonic disease (L2) and ileocolonic disease (L3).

Variable	L1 ($n = 20$)	L2 ($n = 7$)	L3 ($n = 47$)
Mild Clinical Activity	8 (40%)	3 (43%)	15 (32%)
Moderate Clinical Activity	12 (60%)	3 (43%)	30 (64%)
Severe Clinical Activity	0	1 (14%)	2 (4%)

p value = 0.739.

Table S2. Distribution of the clinical activity of disease at baseline in Crohn's disease patients with inflammatory behaviour (B1), stricturing behaviour (B2) and penetrating behaviour (B3).

Variable	B1 ($n = 24$)	B2 ($n = 23$)	B3 ($n = 27$)
Mild Clinical Activity	12 (50%)	1 (4%)	13 (48%)
Moderate Clinical Activity	10 (42%)	22 (96%)	13 (48%)
Severe Clinical Activity	2 (8%)	0	1 (4%)

Association between moderate clinical activity and stricturing behaviour, $p = 0.001$.

Table S3. Distribution of the clinical activity of disease at baseline in patients with distal/left-sided colitis or extensive ulcerative colitis.

Variable	UC patients with distal/left-sided colitis ($n = 40$)	UC patients with extensive colitis ($n = 67$)	X^2
Mild Clinical Activity	13 (32%)	18 (27%)	$p = 0.534$
Moderate Clinical Activity	24 (60%)	40 (60%)	$p = 0.975$
Severe Clinical Activity	3 (8%)	9 (13%)	$p = 0.346$

Table S4. Distribution of baseline characteristics in Crohn's disease patients with clinical remission (remitters) as compared to patients without clinical response (non-responders) at week 14.

**Variable**	**CD remitters (*n* = 17)**	**CD non-responders (*n* = 40)**	**$X^{2/}$ Fisher test**
male gender, *n* (%)	11 (65%)	15 (37%)	$p = 0.059$
young age at diagnosis (<40 years), *n* (%)	13 (76%)	33 (82%)	$p = 0.597$
long standing disease (≥8 years), *n* (%)	16 (94%)	29 (72%)	$p = 0.067$
perianal disease, *n* (%)	6 (35%)	12 (30%)	$p = 0.694$
prior ileo-colonic resection, *n* (%)	12 (71%)	25 (62%)	$p = 0.558$
smoking status, *n* (%)			
Never	10 (59%)	15 (37%)	$p = 0.137$
Former	3 (18%)	13 (32%)	$p = 0.253$
Current	4 (23%)	12 (31%)	$p = 0.618$
prior immunosuppressive therapy, *n* (%)	11 (65%)	32 (80%)	$p = 0.219$
prior TNF antagonist, *n* (%)	13 (76%)	35 (87%)	$p = 0.296$
concomitant steroids during the induction, *n* (%)	8 (46%)	21 (52%)	$p = 0.706$
concomitant immunosuppressive therapy during the induction, *n* (%)	1 (6%)	4 (10%)	$p = 0.615$
mild clinical activity (HBI 5–7), *n* (%)	11 (65%)	7 (18%)	$\boldsymbol{p = 0.0004}$
moderate clinical activity (HBI 8–16), *n* (%)	6 (35%)	31 (77%)	$\boldsymbol{p = 0.002}$
severe disease activity (HBI >16), *n* (%)	0	2 (5%)	$p = 1$
CRP >5 mg/L, *n* (%)	9 (53%)	23 (57%)	$p = 0.751$

Table S5. Distribution of baseline characteristics in Ulcerative colitis patients with clinical remission (remitters) as compared to patients without clinical response (non-responders) at week 14.

**Variable**	**UC remitters (*n* = 34)**	**UC non-responders (*n* = 47)**	X^2
male gender, *n* (%)	21 (62%)	24 (51%)	$p = 0.338$
young age at diagnosis (<40 years), *n* (%)	25 (73%)	34 (72%)	$p = 0.905$
long standing disease (≥8 years), *n* (%)	25 (73%)	35 (74%)	$p = 0.924$
smoking status, *n* (%)			
never	25 (73%)	33 (70%)	$p = 0.743$
former	5 (15%)	9 (19%)	$p = 0.601$
current	4 (12%)	5 (11%)	$p = 0.873$
prior immunosuppressive therapy, *n* (%)	25 (73%)	33 (70%)	$p = 0.743$
prior TNF antagonist, *n* (%)	27 (79%)	37 (79%)	$p = 0.940$
concomitant steroids during the induction, *n* (%)	22 (65%)	31 (66%)	$p = 0.906$
concomitant immunosuppressive therapy during the induction, *n* (%)	3 (9%)	2 (4%)	$p = 0.399$
mild clinical activity (pMayo 2–4), *n* (%)	13 (38%)	14 (30%)	$p = 0.426$
moderate clinical activity (pMayo 5–7), *n* (%)	21 (62%)	24 (51%)	$p = 0.338$
severe disease activity (pMayo 8–9), *n* (%)	0	9 (19%)	$\boldsymbol{p = 0.008}$
CRP >5 mg/L, *n* (%)	20 (59%)	28 (59%)	$p = 0.945$
Distal/Left-sided colitis	21 (62%)	9 (19%)	$\boldsymbol{p = 0.00008}$
Extensive Colitis	13 (38%)	38 (81%)	$\boldsymbol{p = 0.00008}$

Table S6. Distribution of baseline characteristics in Crohn's disease patients with and without clinical response at week 14.

**Variable**	**CD responders (n = 17)**	**CD non-responders (n = 40)**	X^2
male gender, *n* (%)	8 (47%)	15 (37%)	$p = 0.500$
young age at diagnosis (<40 years), *n* (%)	16 (94%)	33 (82%)	$p = 0.247$
long standing disease (≥8 years), *n* (%)	14 (82%)	29 (72%)	$p = 0.429$
perianal disease, *n* (%)	5 (29%)	12 (30%)	$p = 0.964$
prior ileo-colonic resection, *n* (%)	7 (41%)	25 (62%)	$p = 0.137$
smoking status, *n* (%)			
Never	9 (53%)	15 (37%)	$p = 0.280$
Former	3 (18%)	13 (32%)	$p = 0.253$
Current	5 (29%)	12 (31%)	$p = 0.964$
prior immunosuppressive therapy, *n* (%)	14 (82%)	32 (80%)	$p = 0.836$
prior TNF antagonist, *n* (%)	15 (88%)	35 (87%)	$p = 0.938$
concomitant steroids during the induction, *n* (%)	9 (53%)	21 (52%)	$p = 0.975$
concomitant immunosuppressive therapy during the induction, *n* (%)	5 (29%)	4 (10%)	$p = 0.065$
mild clinical activity (HBI 5–7), *n* (%)	6 (35%)	7 (18%)	$p = 0.142$
moderate clinical activity (HBI 8–16), *n* (%)	10 (59%)	31 (77%)	$p = 0.151$
severe disease activity (HBI > 16), *n* (%)	1 (6%)	2 (5%)	$p = 0.891$
CRP > 5mg/L, *n* (%)	12 (71%)	23 (57%)	$p = 0.353$

Table S7. Distribution of baseline characteristics in Ulcerative colitis patients with and without clinical response at week 14.

Variable	UC responders (n = 26)	UC non-responders (n = 47)	X^2
male gender, n (%)	11 (42%)	24 (51%)	p = 0.473
young age at diagnosis (<40 years), n (%)	16 (62%)	34 (72%)	p = 0.341
long standing disease (≥8 years), n (%)	20 (77%)	35 (74%)	p = 0.815
smoking status, n (%)			
never	16 (62%)	33 (70%)	p = 0.449
former	5 (19%)	9 (19%)	p = 0.993
current	5 (19%)	5 (11%)	p = 0.306
prior immunosuppressive therapy, n (%)	18 (69%)	33 (70%)	p = 0.930
prior TNF antagonist, n (%)	22 (85%)	37 (79%)	p = 0.540
concomitant steroids during the induction, n (%)	16 (62%)	31 (66%)	p = 0.705
concomitant immunosuppressive therapy during the induction, n (%)	4 (15%)	2 (4%)	p = 0.097
mild clinical activity (pMayo 2–4), n (%)	4 (15%)	14 (30%)	p = 0.171
moderate clinical activity (pMayo 5–7), n (%)	19 (73%)	24 (51%)	p = 0.067
severe disease activity (pMayo 8–9), n (%)	3 (12%)	9 (19%)	p = 0.400
CRP >5 mg/L, n (%)	13 (50%)	28 (59%)	p = 0.429
Distal/Left-sided colitis	10 (38%)	9 (19%)	p = 0.071
Extensive Colitis	16 (62%)	38 (81%)	p = 0.071
